# Gorham-Stout syndrome: A chylothorax disease with bony destruction: A case report

**DOI:** 10.1097/MD.0000000000032105

**Published:** 2022-12-16

**Authors:** Ping-Yang Hong, Xiao-Bin Zhang, Hui-Qing Zeng, Yi-Lin Zhao, Mao-Hong Huang

**Affiliations:** a Department of Pulmonary and Critical Care Medicine, Zhongshan Hospital of Xiamen University, School of Medicine, Xiamen University; the Third Clinical Medical College of Fujian Medical University, Fujian, China; b Department of Oncology and Vascular Intervention, Zhongshan Hospital of Xiamen University, School of Medicine, Xiamen University; the Third Clinical Medical College of Fujian Medical University, Fujian, China.

**Keywords:** chylothorax, Gorham–Stout syndrome, osteolysis, zoledronic acid

## Abstract

Gorham–Stout syndrome is a sporadic condition characterized by a tumor-like lesion with extensive osteolysis, severe symptoms, and a poor prognosis. Poor prognostic indicators include osteolytic lesions of the spine and pleura effusion.

**Patient concerns:**

A 67-year-old Chinese man with five months history of chest tightness presented to our institution with aggravated shortness of breath. Ultrasonography demonstrated hydrothorax on the right side. The patient's imaging studies (computerized tomography [CT] scan, magnetic resonance imaging, and positron emission tomography [PET]/CT) revealed osteolytic lesions (the skull, several spines, several ribs, both shoulder blades, and the pelvis).

**Diagnoses:**

Gorham-Stout syndrome. (4) Interventions: We advised the patient to follow a low-fat diet. On the patient, we performed a superior vena cava angiography. The injection of zoledronic acid was used to prevent bone loss.

**Outcomes:**

We found resolution of chylothorax after a low-fat diet, superior vena cava angiography and injection of zoledronic acid.

**Lessons:**

The possibility of Gorham -Stout syndrome should be ruled out in patients with clinical chylothorax. The relief of chylothorax requires comprehensive treatment.

## 1. Introduction

Gorham–Stout syndrome is a sporadic condition characterized by a tumor-like lesion with extensive osteolysis, severe symptoms, and a poor prognosis. Poor prognostic indicators include osteolytic lesions of the spine and pleural effusion.^[1]^ The involvement of certain bones, such as ribs, and scapula, can cause chylothorax and are considered negative prognostic factors because life-threatening complications can occur.^[2]^ There is currently no cure for chylothorax in these patients.^[3]^ After a low-fat diet, superior vena cava angiography and zoledronic acid injection, regression of chylothorax was observed. However, the mechanism of chylothorax improvement requires more research.

## 2. Case presentation

**Figure 1.**
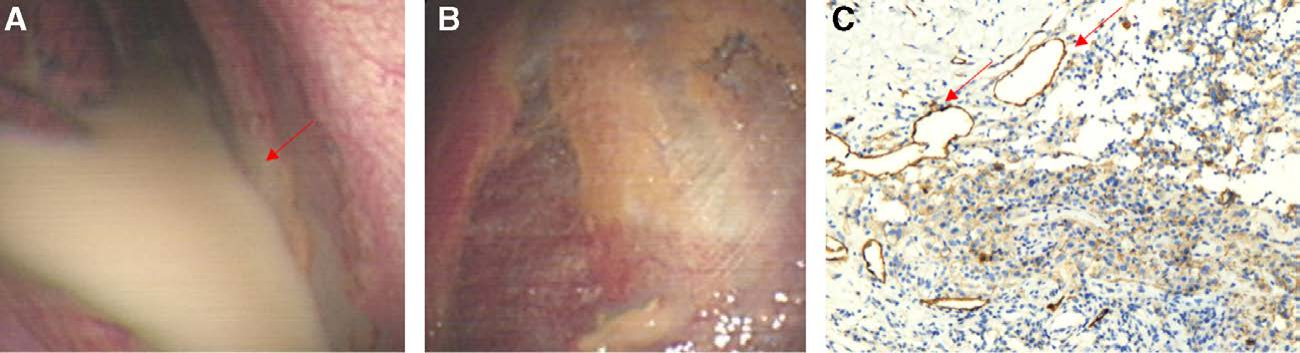
(A): Chylothorax. (B): Thoracoscopic pleural findings were normal. (C): Postoperative pathology confirmed that lymphatic-derived endothelial cells of pleura were positive for D2-40 staining.

**Figure 2.**
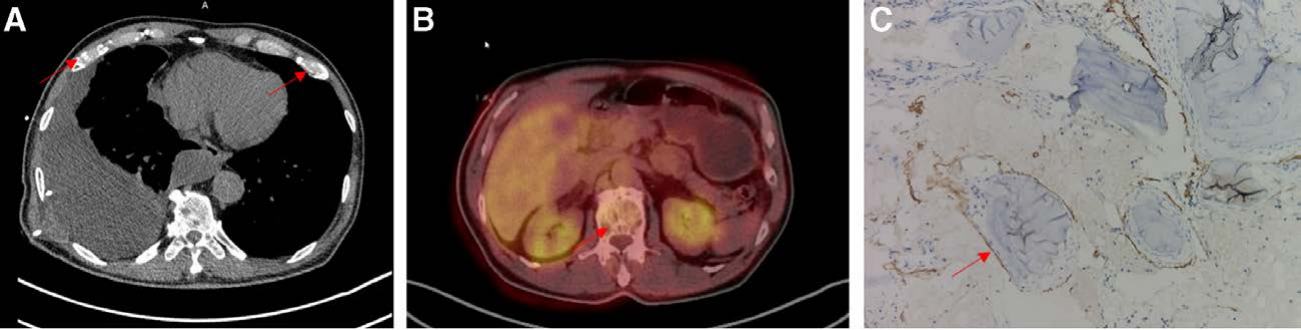
(A): CT scan demonstrated massive bony destruction of several spines. (B): Positron emission tomography-computed tomography demonstrated massive bony destruction of several ribs. (C): Postoperative pathology confirmed that part of the bone was replaced by lymphatic-derived endothelial cells, which were positive for D2-40 staining. CT = computerized tomography.

A 67-year-old Chinese man with 5 months history of chest tightness presented to our institution with aggravated shortness of breath. Ultrasonography demonstrated hydrothorax on the right side. Thoracentesis was performed, and the test results confirmed chylothorax (Fig. 1) (lactate dehydrogenase, 143.8 U/L; glucose, 5.92 mmol/L; protein, 48.7 g/L; adenosine deaminase, 8.3 U/L, positive chylous test [Sudan III staining]; cholesterol, 0.8 mmol/L; triglycerides, 9.2 mmol/L; negative cultures; cell count with 98% mononuclear cells and negative biopsy for cancer; total protein and lactate dehydrogenase of blood, 72.4 g/L and 195.4 U/L). A computed tomographic scan and positron emission tomography-computed tomography demonstrated extensive bony destruction of the skull, several spines, several ribs, scapulas, and the pelvis (Fig. 2).

The tumor markers carbohydrate antigen (CA) 125, CA 19-9 value, CA15-3, CA 72-4, carcinoembryonic antigen, squamous cell carcinoma antigen, neuron-specific enolase (NSE), alpha-fetoprotein, and prostate-specific antigen had values within the normal range. Average values of serological markers of autoimmunity, creatine kinase-MB, rheumatoid factor, antithyroglobulin antibody, and circulating immune complexes excluded autoimmunity. The markers of myocardial injury and liver and kidney function were normal. The indicators associated with tuberculosis were normal. The laboratory tests showed no increase in inflammation markers. No signs of tumor were found by thoracoscopy (Fig. 1), gastroscopy, and colonoscopy. No particular relevant circumstances regarding his family history were identified. A tissue biopsy of the spine was performed, and the postoperative pathology confirmed that part of the bone was replaced by lymphatic-derived endothelial cells, which were positive for D2-40 staining (Fig. 2).

**Figure 3.**
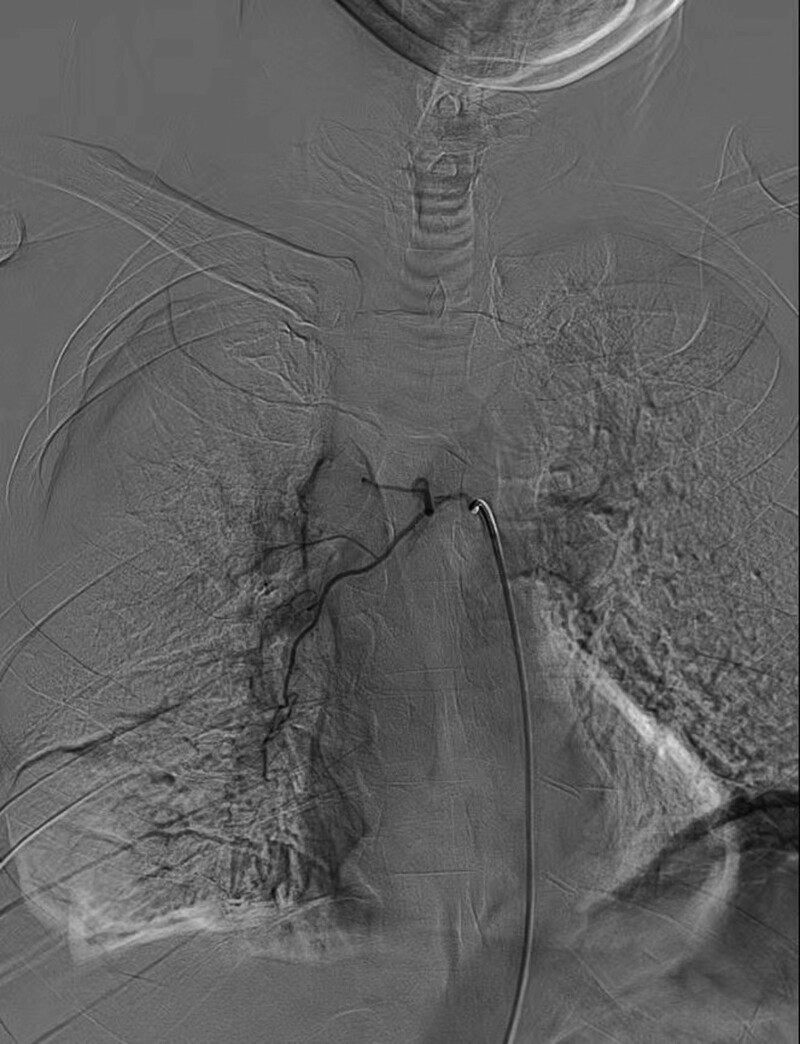
Abnormal enlargement of azygos vein into superior vena cava and local contrast agent retention was observed.

The diagnosis of Gorham-Stout disease^[1]^ was established based on the clinical and imaging elements and the exclusion of pathologies such as parasitosis, thyroid or parathyroid disease, parasitosis, lymphoma, neoplasia, and autoimmune disorder. We performed a superior vena cava angiography on the patient. Abnormal enlargement of the azygos vein into the superior vena cava and local contrast agent retention was observed (Fig. 3). We instructed our patients to follow a low-fat diet in treating chylothorax. The zoledronic acid injection (4 mg each time, once every 3 weeks) was used to inhibit bone destruction. His shortness of breath was relieved significantly after the comprehensive therapy. At the 2-month follow-up visit, he reported continuous shortness of breath relief.

## 3. Discussion and conclusions

Gorham–Stout syndrome, characterized by a tumor-like lesion with large osteolysis, is sporadic with severe symptoms and poor prognosis.^[1,2]^ Local swelling can occur when the lesion invades the soft tissue surrounding the bone, and chylothorax can occur when the lesion invades the chest wall, ribs, vertebrae, or thoracic duct.^[4]^ Gorham–Stout syndrome’s etiology and pathogenesis remain uncertain and controversial.^[5]^ The existence and role of osteoclasts and vascular and lymphatic vessels have been controversial in the pathogenesis of Gorham–Stout syndrome.^[6–9]^

Clinical manifestations of Gorham–Stout syndrome vary, with onset ages ranging from 1 month to 92 years. The severity of the disease usually depends on the complicated structures and the progression of osteolysis, and there are generally no systemic symptoms.^[10,11]^ Past trauma has been mentioned in specific individuals.^[12]^ There was no history of trauma in our case, and the disease began after age 65.

In Gorham- Stout syndrome, localized discomfort, swelling, weakness, functional impairment of afflicted limbs, respiratory distress and failure, neurological problems, deformities, and paralysis are joint. Gorham–Stout syndrome commonly affected areas include the ribs, spine, pelvis, limb bones, and shoulder blades.^[13]^ This case was characterized by progressive dyspnea, and the symptoms caused by bone destruction were not obvious.

In addition to conventional X-ray, CT, and magnetic resonance imaging, bone scan, single photon emission computed tomography/CT, and PET/CT can also be used for imaging examination of Gorham–Stout syndrome.^[4]^ X-rays usually show bone absorption but no periosteal reaction. CT scan can show the location and number of osteolytic lesions more clearly than X-ray.^[14]^ 18F-NaF-PET/CT showed higher consistency in Gorham–Stout syndrome lesions, opening new possibilities for diagnosis, degree of disease activity, and treatment response.^[15]^ Our case contains multiple bone lesions consistent with the typical presentation of Gorham–Stout syndrome.

Pathological diagnosis is essential for the diagnosis of Gorham–Stout syndrome. The pathological findings are closely related to the site of the specimen. The typical pathological findings are bone resorption and replacement by vascular or lymphatic thin-walled monolayer endothelial cells, accompanied by infiltration of lymphatic and vascular soft tissue without cellular atypia and inflammatory infiltration.^[4]^ Immunohistochemistry shows that D2-40 intense staining of lymphatic endothelial cells in dilated lymphatic vessels may be one of the characteristics of Gorham–Stout syndrome,^[16]^ which is consistent with the pathological findings of our case.

There are no biomarkers for Gorham–Stout syndrome; however, several studies have mentioned miRNAs and serum biomarkers as potential biomarkers.^[2,17]^

Given the disease’s rarity, there are no established treatment guidelines. However, pharmaceutical therapy, surgery, radiotherapy, or combinations have been studied. Interferon, bisphosphonates, calcium salts, and vitamin D; interferon and zoledronic acid; cyclophosphamide and fluorouracil; salmon calcitonin, alendronate sodium, and sirolimus were among the pharmacological therapies employed.^[4,18]^ Sirolimus is an inhibitor of the mammalian target of rapamycin, which has antiangiogenic properties and prevents the development of chylothorax in Gorham–Stout syndrome patients.^[19]^ The chylothorax did not develop in our case. The reason may be that the contrast agent blocks abnormal lymphatic vessels. Alternatively, maybe the low-fat diet played a role. Most likely, zoledronic acid improved the disease situation. A study has shown that Gorham–Stout syndrome, including chylothorax, may be well controlled with zoledronic acid.^[20]^

The main manifestations of our patient were chylothorax and bone destruction. After completing many examinations, the etiology was still unknown. The diagnosis was confirmed by thoracoscopy and bone biopsy. Therefore, the possibility of Gorham–Stout syndrome should be ruled out in patients with clinical chylothorax. After comprehensive treatment, the progress of chylothorax was relieved. More research is needed on the treatment of chylothorax in Gorham–Stout syndrome patients.

## Author contributions

Conception and design: P-Y Hong, X-B Zhang, and Y-L Zhao. Collection and assembly of data: M-H Huang and H-Q Zeng. Data analysis and interpretation: X-B Zhang and M-H Huang. Manuscript writing: All authors. Final approval of manuscript: All authors.

**Data curation:** Hui-Qing Zeng, Xiao-Bin Zhang.

**Formal analysis:** Xiao-Bin Zhang.

**Funding acquisition:** Xiao-Bin Zhang.

**Investigation:** Ping-Yang Hong.

**Methodology:** Ping-Yang Hong, Yi-Lin Zhao.

**Supervision:** Mao-Hong Huang, Xiao-Bin Zhang.

**Writing – review & editing:** Ping-Yang Hong, Xiao-Bin Zhang.
